# Translational learning from clinical studies predicts drug pharmacokinetics across patient populations

**DOI:** 10.1038/s41540-017-0012-5

**Published:** 2017-03-28

**Authors:** Markus Krauss, Ute Hofmann, Clemens Schafmayer, Svitlana Igel, Jan Schlender, Christian Mueller, Mario Brosch, Witigo von Schoenfels, Wiebke Erhart, Andreas Schuppert, Michael Block, Elke Schaeffeler, Gabriele Boehmer, Linus Goerlitz, Jan Hoecker, Joerg Lippert, Reinhold Kerb, Jochen Hampe, Lars Kuepfer, Matthias Schwab

**Affiliations:** 1 Systems Pharmacology, Bayer AG, Leverkusen, 51368 Germany; 2 Dr. Margarete Fischer-Bosch Institute of Clinical Pharmacology, University of Tuebingen, Stuttgart, 70376 Germany; 3 Department of General Surgery and Thoracic Surgery, University Hospital Schleswig-Holstein, Kiel, 24105 Germany; 4 Applied Mathematics, Bayer AG, Leverkusen, 51368 Germany; 5 Department of Medicine I, University Medical Center Dresden, Technical University Dresden, Dresden, 01307 Germany; 6 Technology Development, Bayer AG, Leverkusen, 51368 Germany; 7 Joint Research Center for Computational Biomedicine, RWTH Aachen University, Aachen, 52074 Germany; 8 Department of Clinical Pharmacology, University Hospital Tuebingen, Tuebingen, 72076 Germany; 9 Clinical Pharmacometrics, Bayer Pharma AG, Berlin, 13353 Germany; 10 Department of Pharmacy and Biochemistry, University of Tuebingen, Tuebingen, 72074 Germany

## Abstract

Early indication of late-stage failure of novel candidate drugs could be facilitated by continuous integration, assessment, and transfer of knowledge acquired along pharmaceutical development programs. We here present a translational systems pharmacology workflow that combines drug cocktail probing in a specifically designed clinical study, physiologically based pharmacokinetic modeling, and Bayesian statistics to identify and transfer (patho-)physiological and drug-specific knowledge across distinct patient populations. Our work builds on two clinical investigations, one with 103 healthy volunteers and one with 79 diseased patients from which we systematically derived physiological information from pharmacokinetic data for a reference probe drug (midazolam) at the single-patient level. Taking into account the acquired knowledge describing (patho-)physiological alterations in the patient cohort allowed the successful prediction of the population pharmacokinetics of a second, candidate probe drug (torsemide) in the patient population. In addition, we identified significant relations of the acquired physiological processes to patient metadata from liver biopsies. The presented prototypical systems pharmacology approach is a proof of concept for model-based translation across different stages of pharmaceutical development programs. Applied consistently, it has the potential to systematically improve predictivity of pharmacokinetic simulations by incorporating the results of clinical trials and translating them to subsequent studies.

## Systems pharmacology: predicting population pharmacokinetics in silico

Physiologically based modeling together with Bayesian statistics allows the prediction of drug pharmacokinetics in specific patient populations. An interdisciplinary group of clinicians and computational scientists led by Dr. Lars Kuepfer from Bayer developed a generic workflow consisting of several consecutive learning steps where knowledge about both individual physiology as well as drug physicochemistry can be efficiently derived from plasma concentration profiles. The acquired information is then be used for the prediction of the pharmacokinetic behavior of a new drug candidate in a diseased population. This allows to simulate the variability in drug exposure virtually before starting clinical investigation in real patients in order to evaluate drug safety or efficacy through the simulation of virtual populations. Further development of this workflow could improve the safety of clinical development programs to assess the risk-benefit ratio of novel drug candidates in silico.

## Introduction

Knowledge management is a key challenge in any pharmaceutical development program. It is a plausible hope that a large part of today’s limitations in pharmaceutical research will be addressed by knowledge-based translation, curation, and continuous re-evaluation of information and data along the developmental path of novel medicines. Thus, the aim of translational systems pharmacology approaches is to provide support in (i) translation of preclinical findings from animal models to human healthy volunteers, (ii) bridging between healthy volunteers and patients, and (iii) revision of discovery objectives in clinical research programs.^1–5^ Another important goal of translational systems pharmacology is a better understanding of variability in drug behavior and drug action between individuals in a specific patient cohort. The mechanistic explanation of such observations in clinical practice would be a particularly valuable outcome since it might help to identify potential subgroups of non-responders or the occurrence of adverse drug events in high-risk subgroups of patients.^1, 6, 7^

The main challenge for information management in pharmaceutical research is the extraction of information from clinical raw data in a format that is accessible for translational tasks, ideally in a continuously growing integrated information repository.^6, 8, 9^ Physiologically based pharmacokinetic (PBPK) models represent a possibility to address this need, since they allow the integration of experimental data from different layers of biological organization PBPK models are compartmental ordinary differential equation-based models for a mechanistic description of physiological processes underlying drug ADME (ADME: absorption, distribution, metabolization, and excretion).^10, 11^ As such, PBPK models are particularly well suited for comprehensive analyses of drug pharmacokinetics (PK) and, furthermore, for translational approaches.^12^ Before, PBPK modeling has been used successfully among others for drug–drug interaction studies,^13^ pediatric scaling,^14, 15^ patient stratification,^16, 17^ and risk-assessment.^7, 18^ Today, PBPK models are routinely used in pharmaceutical development programs and they are increasingly accepted by regulatory authorities.^19–22^

The goal of the present study is to translate physiological information from healthy volunteers to obese patients, with the aim of predicting the PK of a candidate drug in the obese cohort. For that, we introduce a translational systems pharmacology workflow (henceforth referred to as translational approach). The concept aims for an iterative application of a previously established Bayesian population PBPK approach (henceforth referred to as Bayesian-PBPK analysis),^23, 24^ which allows to acquire the functional origins of interindividual variability in the physiology of patient subgroups. Furthermore, we compare the acquired knowledge to patient metadata for further analyses of pathophysiological alterations in the diseased cohort. Altogether this proof-of-concept study provides strong evidence for the impact of systems pharmacology to improve drug development and translational science.

## Results

### Translational approach

**Fig. 1:**
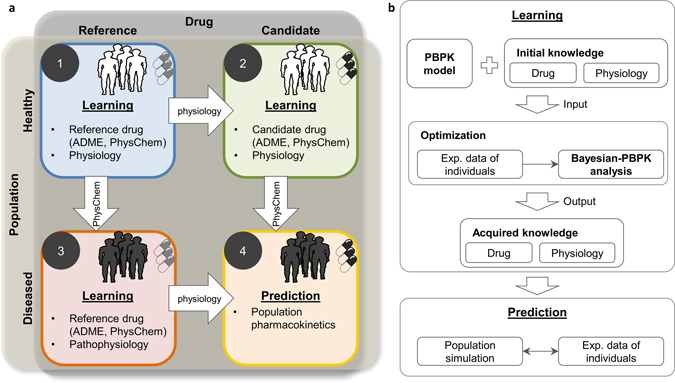
Schematic illustration of the translational approach. **a** A learning step contains a full Bayesian analysis where initial knowledge is used in combination with new experimental data to refine and acquire knowledge about physiological and drug-specific parameters. A translation step transfers the acquired knowledge to a new investigation where the acquired knowledge is used as initial knowledge in a new Bayesian analysis. In this illustration, learning starts from the healthy population treated with a reference drug and ultimately leads to prediction of the effects of a candidate drug in a diseased population. **b** The presented learning scheme is performed in each step of the translational learning workflow. The central element is the Bayesian-PBPK analysis. Initial knowledge is updated with new experimental data, and acquired knowledge on both the drug and population physiology is inferred. Assessed knowledge can then be used for the pharmacokinetic prediction of a drug in the population of interest and subsequently be compared with experimental data

The presented translational approach consists of three learning steps and a subsequent prediction step (Fig. 1). In each learning step, a Bayesian-PBPK analysis extracts knowledge about physiological and physicochemical parameters from experimental data, taking into account available initial information about corresponding parameters in the PBPK model. Translation of the acquired knowledge is possible due to the mechanistic representation of information in PBPK models, as the underlying structure allows to transfer assessed parameter distributions as initial knowledge in the subsequent Bayesian-PBPK analyses.

In particular, the four steps of the translational approach are as follows: in step one, a Bayesian-PBPK analysis is performed using study data of the reference probe drug midazolam in a cohort of 20 randomly chosen healthy volunteers (phase I study). In step two, the physiological knowledge acquired in step one is refined in combination with study data of the candidate probe drug torsemide in the identical 20 healthy volunteers. Furthermore, the physicochemistry of the candidate drug is identified. In step three, the physicochemical knowledge of midazolam acquired in step one is used with study data of 20 randomly chosen obese individuals out of the diseased patient cohort (phase II study) to identify the pathophysiological changes in this population. Finally, the acquired physicochemistry of torsemide from step two and the assessed pathophysiology from step three are combined for a de novo prediction of torsemide population PK in the obese cohort in step four. Notably, the Bayesian-PBPK analyses generate individual-specific information in the three learning steps and simultaneously allow to quantify the population-specific interindividual variability (see “Material and methods”).

### Evaluation of the clinical study

The translational approach proposed here was applied to a clinical study, conducted within the Virtual Liver program.^25, 26^ The study involved 103 healthy volunteers and 79 diseased patients scheduled for liver biopsy and visceral surgery. Both cohorts received the same cocktail of six approved and commonly used drugs (midazolam, torsemide, talinolol, pravastatin, codeine, and caffeine) at sub-therapeutic doses.^26^ For conceptual illustration of our translational approach, we focused on the PK behavior of midazolam and torsemide.

The anthropometric features of the individuals and patients are summarized in Table 1. As expected, the main difference between the two cohorts was body weight; the median values were 74.5 and 138.0 kg, respectively, which is in line with previous studies and the high percentage of obesity (72%, Table S1) in the diseased patient cohort.^27, 28^

**Table 1:** Summary of statistics for anthropometrical parameters in both populations

	*n*	Male (#)	Age (years)		Body weight (kg)		Body height (m)		Body mass index [kg/m^2^]	
			Median	[Min max]	Median	[Min max]	Median	[Min max]	Median	[Min max]
Healthy individuals	103	54	28	[18 56]	74.5	[48.5 113]	1.74	[1.54 1.94]	23.5	[18.8 32.3]
Diseased patients	79	33	45	[20 77]	138	[52 206]	1.75	[1.56 1.92]	47.3	[19.7 67.1]

**Fig. 2:**
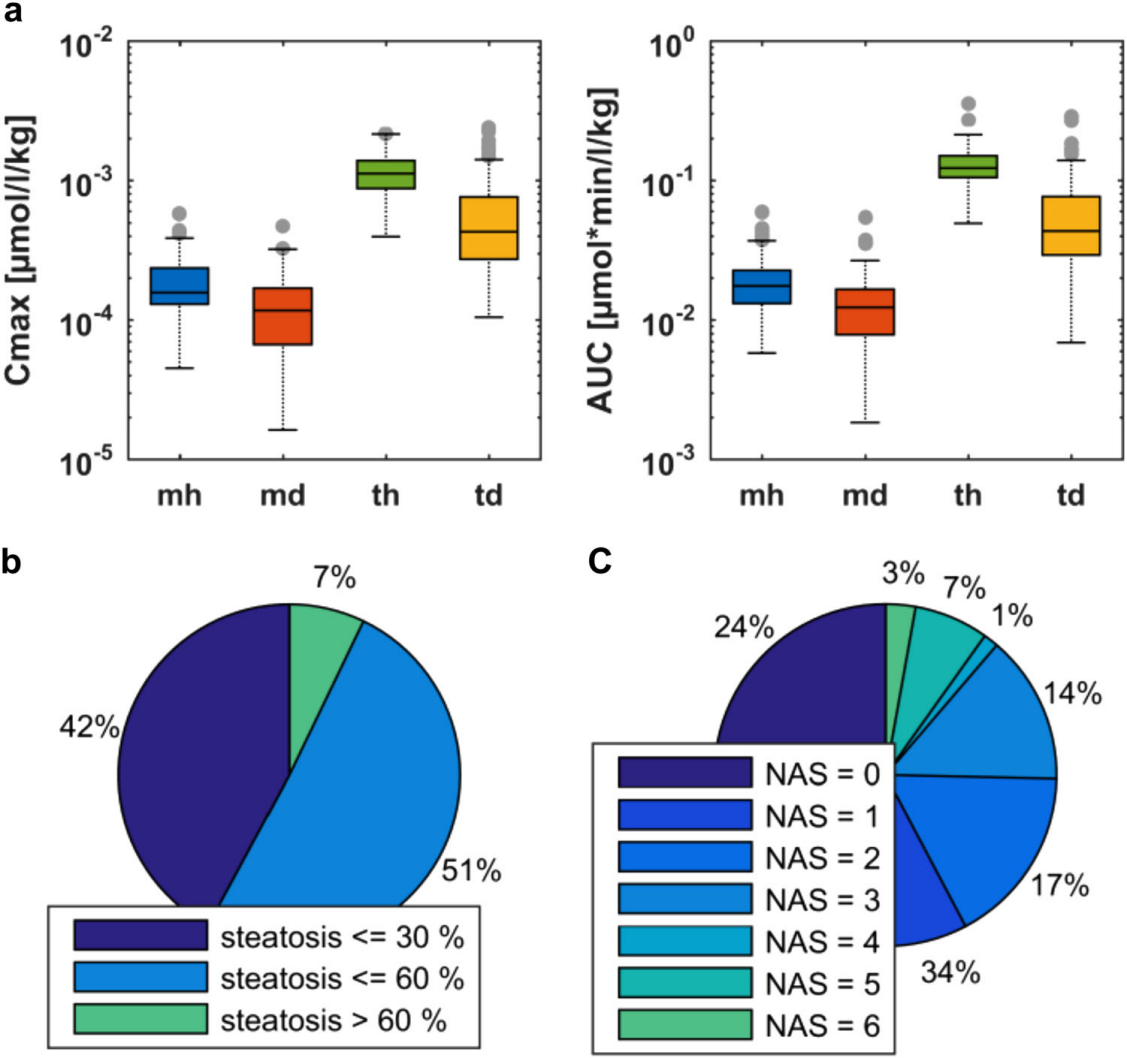
Evaluation of experimental PK data and individual metadata for midazolam and torsemide in healthy individuals and diseased patients. **a** Boxplots of Cmax and AUC for healthy individuals and diseased patients treated with midazolam or torsemide. **b** Fractions of individuals with different levels of steatosis. **c** Fractions of individuals with different NAS. *Blue* indicates midazolam data from healthy population (mh); *green* torsemide data from healthy population (th); *red* midazolam data from diseased population (md); *yellow* torsemide data from diseased population (td)

Statistics for peak concentration (Cmax) and area under the curve (AUC) are summarized in Table S2. Due to the negative correlation of body weight and the PK parameters, values are normalized to individual weight (Fig. S1A). Significant differences between the healthy and diseased populations were obtained in the cases of both midazolam and torsemide, as illustrated in Fig. 2a. Our initial analysis also revealed some statistical outliers in both populations, which could partly be explained by specific clinical metadata for these individuals. In the healthy population, one PK outlier with high AUC for torsemide and low Cmax for OH-torsemide is caused by a homozygous variant genotype for the cytochrome P450 2C9 (CYP2C9)*3 allele, which results in a poor metabolizer phenotype^29^ (Table S3). In the diseased population, all patients identified as outliers with respect to Cmax or AUC were scheduled to undergo a surgery other than bypass of the stomach, due to additional diseases such as cancer (Table S1). However, these patients were neglected for the Bayesian-PBPK analyses.

For the diseased population, correlations revealed significant associations between body weight and steatosis of the hepatocytes. A positive correlation was also obtained for the nonalcoholic fatty liver disease (NAFLD) activity score (NAS)^30^ and body weight of each patient, respectively (Fig. S1B). An analysis of the proportion of individuals with respect to the level of steatosis and to the NAS, respectively (Fig. 2b, c), revealed that only 7% of patients had degenerative steatosis (with more than 60% of the liver affected), but about 25% had a NAS greater than 3. To assess the interindividual variability in each cohort and to further analyze the pathophysiological changes, we next performed the first three steps of the translational approach.

### Individual model simulations

**Fig. 3:**
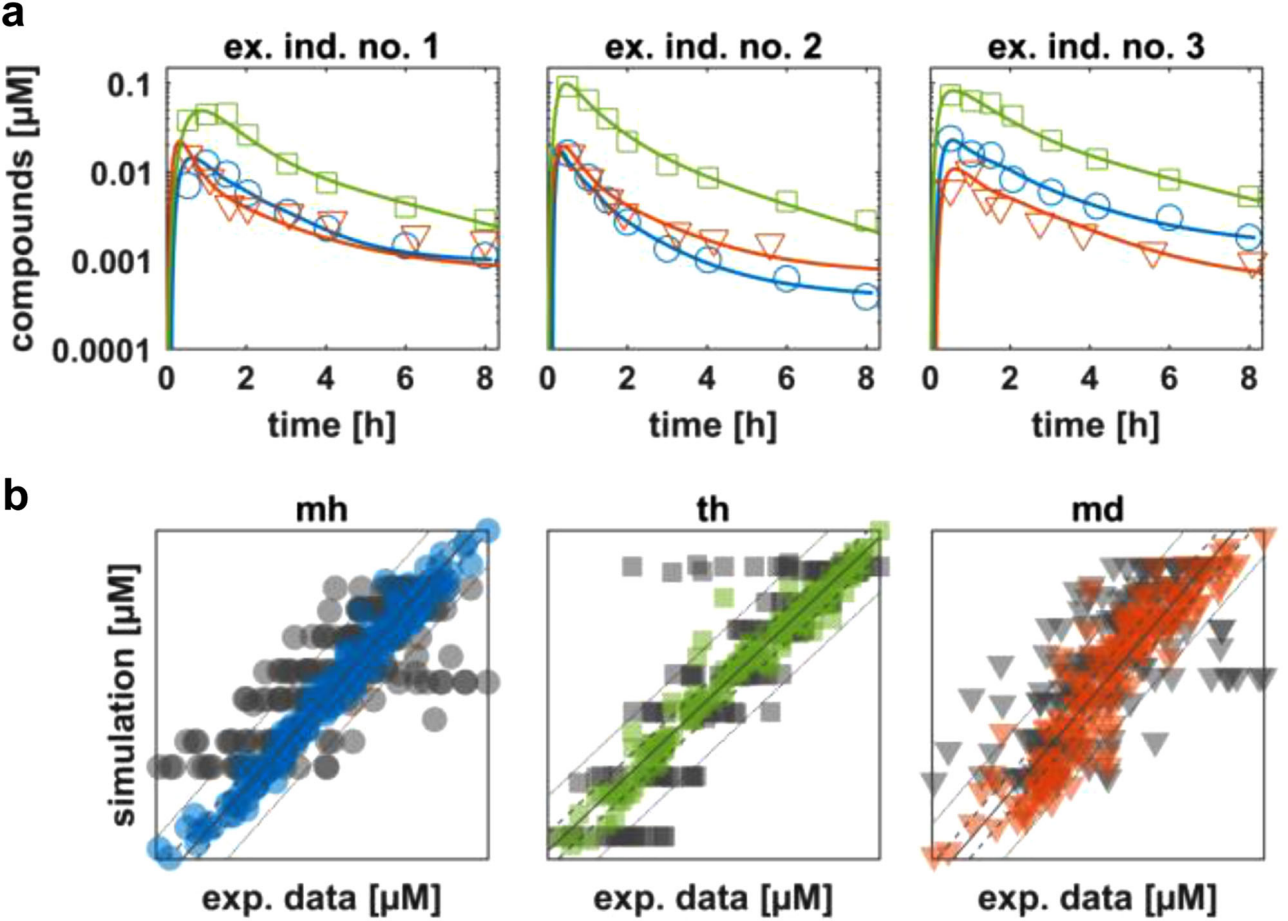
Individual model simulations after application of the three learning steps of the translational approach. **a** Simulations of venous blood plasma concentration based on parameters with maximum posterior probability are shown for three example individuals. **b** Comparison of experimental data of venous blood plasma concentration with simulations of the mean value model (start parameterization) at experimental time points (*gray*), and experimental data from simulations with individual-specific parameterized models (based on acquired distributions) at experimental time points (*colored*). *Blue circles* indicate midazolam data from healthy population (mh); *green squares* indicate torsemide data from healthy population (th); *red triangles* indicate midazolam data from diseased population (md)

As a first qualification of the Bayesian-PBPK analyses, individual PBPK models were developed and simulations were performed for each of the three learning steps. All simulations showed good agreement with the corresponding experimental data for each individual (Figs. S2, S3). This is illustrated in Fig. 3a exemplarily for three individuals. Overall, application of the translational approach resulted in a significantly increased agreement of the experimental data with the simulated PK profiles, relative to the initial knowledge in a mean value PBPK model representing an average patient (Fig. 3b). A comparison of the simulations of the initial state and the simulations after applying the Bayesian-PBPK analyses revealed a decreased root-mean-square-error (RMSE) of weighted residuals by 86% for midazolam and by 70% for torsemide in healthy individuals, respectively, and by 66% for midazolam in the diseased population. In summary, the individual simulations served as an initial validation of the Bayesian-PBPK analyses, and showed that the learning steps of the translational approach were applied successfully.

### Population simulations

**Fig. 4:**
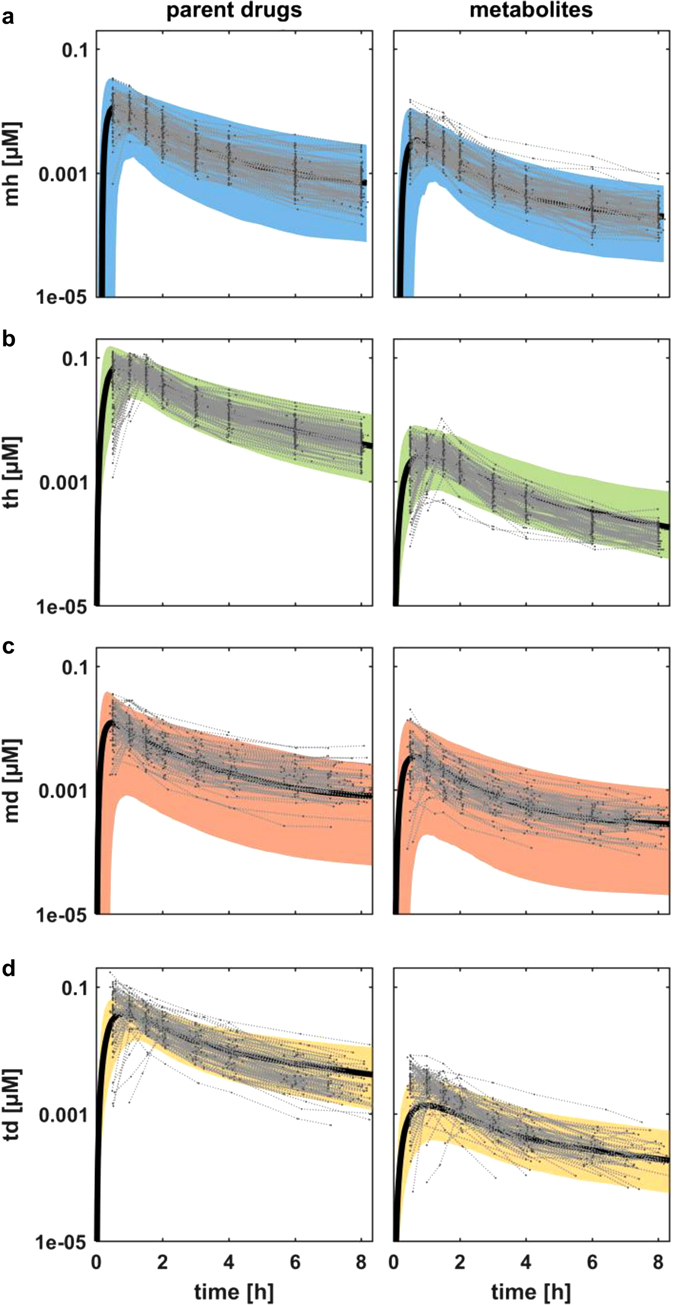
Population simulations and prediction after application of the translational approach. **a**–**c** Simulations of venous blood plasma concentrations **a** of midazolam in the healthy population (mh), **b** of torsemide in the healthy population (th), **c** of midazolam in the diseased population (md). **d** Population PK prediction of torsemide venous blood plasma in the diseased population (td). Shown are the 95% confidence intervals (*colored area*), the mean value curve (*black line*), and the experimental data (*gray dots* connected by *light gray dashed lines*)

The Bayesian-PBPK analyses were next qualified by population simulations. Figure 4a shows the resulting population simulations for midazolam and OH-midazolam, together with the experimental data for the remaining 83 healthy individuals at the original time points. Both simulations are in good agreement with the experimental data, indicating that the population characteristics were accurately identified by step one of the translational approach.

As a qualification of step two of the translational approach, Fig. 4b analogously compares the results of our population simulation of torsemide and OH-torsemide PK to experimental data for the remaining 83 healthy individuals. The population simulation for torsemide is in good agreement to the experimental data. The PK behavior of OH-torsemide is well described during absorption and distribution phases; however, the clearance is slightly overestimated.

After the initial analyses in the healthy population were completed, midazolam PK in the diseased population was investigated in a similar manner. The simulation results were compared with experimental PK data for the 59 remaining diseased patients of the study (Fig. 4c). During the absorption and distribution phases, the PK behavior of midazolam was well described. However, during the terminal phase, the simulation underestimated the experimental data slightly. In contrast, a simulation of the PK behavior of the 1′-OH metabolite of midazolam was in good agreement with the experimental data.

These results served as validation of the acquired knowledge at the population level and, in combination with the simulations of the single individuals, indicate that updating of initial knowledge with the clinical data of healthy individuals and obese patients with the Bayesian-PBPK analysis was effective.

### Pharmacokinetic prediction

The fourth and final step of the described translational approach was prediction of the PK of the candidate probe drug, torsemide, in the cohort of obese patients. Notably, no previous Bayesian-PBPK analysis had been performed for the candidate probe drug in the obese population at this stage. Instead, we translated the acquired knowledge characterizing both the population and the drug from the previous analyses (Fig. 1). Figure 4d shows the resulting prediction for the PK of torsemide and OH-torsemide for an obese population in comparison with measured PK data of all 79 diseased individuals. Overall, the prediction described the data for torsemide well. The absorption phase was slightly underestimated and the terminal phase was, in turn, marginally overestimated. In the case of OH-torsemide, the predicted population simulation underestimated the PK during the absorption phase, but accurately described the PK during the terminal phase.

To assess the quality of the PK prediction of the candidate probe drug, we performed a retrospective Bayesian-PBPK analysis for torsemide in the cohort of 20 obese patients. As before, a population simulation was performed for qualification, thereby using the knowledge acquired from the retrospective analysis. In order to obtain a benchmark for quantitative comparisons, a third population simulation was performed additionally based only on the initial knowledge as in step one. In each case, the normalized RMSE was calculated quantifying the agreement between the median simulation curve and the 79 experimental PK data sets for both torsemide and OH-torsemide (Table 2).

**Table 2:** Quantitative assessment of PK prediction

	Normalized RMSE		
	Initial	Predicted	Retrospective
Torsemide	1	0.457	0.390
OH-torsemide	1	0.691	0.273

The normalized RMSE of the predicted simulation improved by 54% for torsemide and by 31% for OH-torsemide compared with that for the simulation based only on initial information. A further improvement of the simulation was observed in the retrospective analysis, in particular for the terminal phase (Fig. S4). The normalized RMSE for the retrospective analysis was more accurate by 7% for torsemide and 42% for OH-torsemide compared with data of the predicted analysis.

### Acquisition of knowledge

**Fig. 5:**
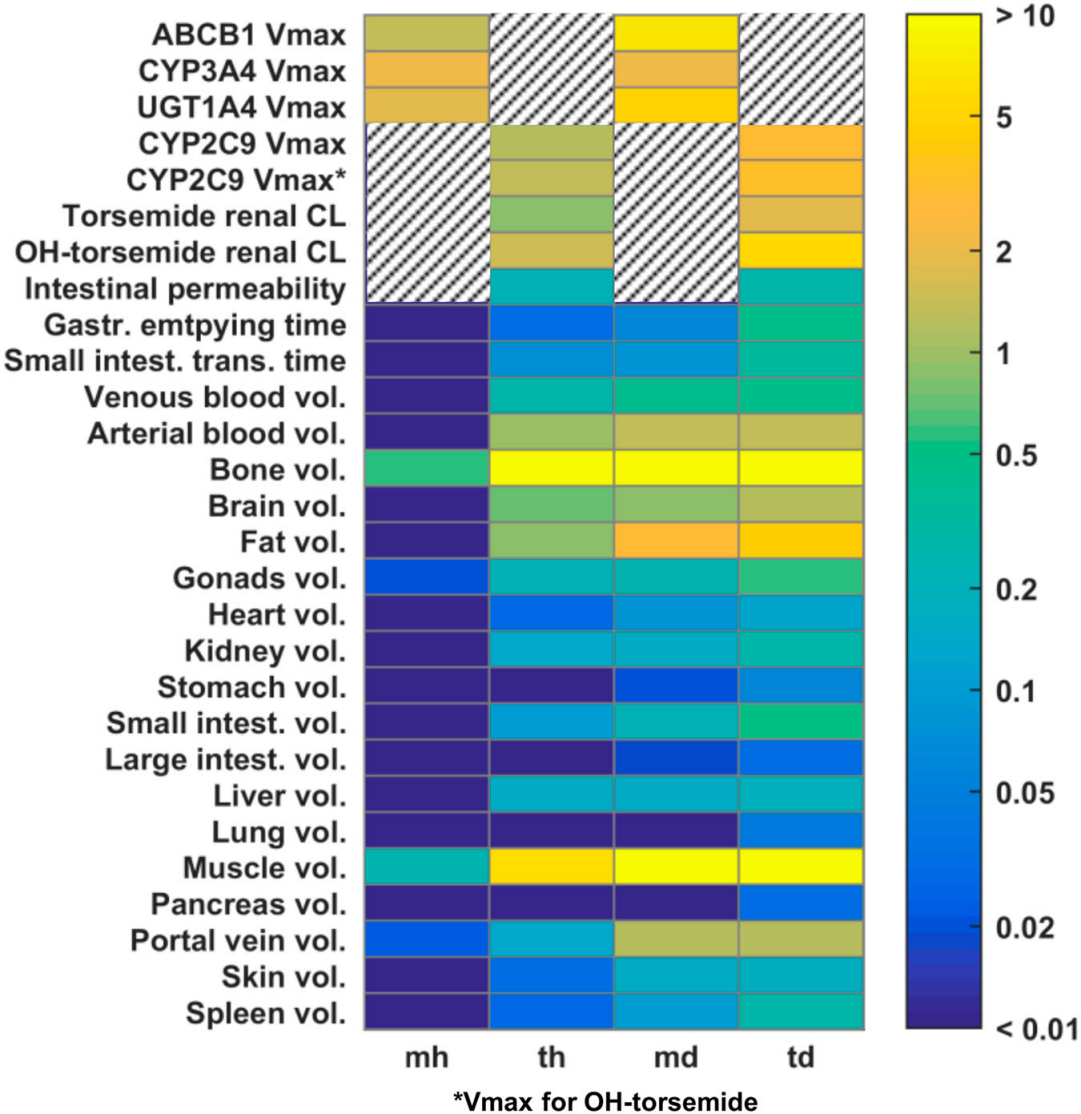
Learning progression. Heat map shows Kullback–Leibler divergence (relative entropy) between acquired knowledge and initial knowledge for each learning step. *Color code* represents learning progression from *blue* (learned nothing) to *yellow* (learned very much). *Hatching* indicates that parameters have not been considered in the respective model such that relative entropy could not be determined

A key question related to the translational approach is which parameters have been most informed by the experimental data, and whether continuous learning can be achieved during the translational approach. The Kullback–Leibler divergence^31^ is a measure of relative entropy and was used to determine the difference in the obtained parameter distribution of specific PBPK model parameters between the acquired and the initial knowledge, respectively, for each learning step (Fig. 5). For example, various active processes characterizing ADME targets in the healthy population showed a large gain of information already in the first steps. Further information was gathered when translating from healthy individuals to obese patients, indicating that a considerable amount of knowledge could be inferred from the experimental data during analysis of the diseased population. Most physiological parameters like organ volumes and blood flow rates (data not shown) revealed only a small gain of information for the analyses in the healthy population compared with the initial knowledge. In contrast, after translation to the obese cohort, a larger information gain was achieved. For further quantification, all parameters for which the geometric mean value changed by more than 10% are shown in Tables S4–S7.

### Characterization of pathophysiological alterations

**Fig. 6:**
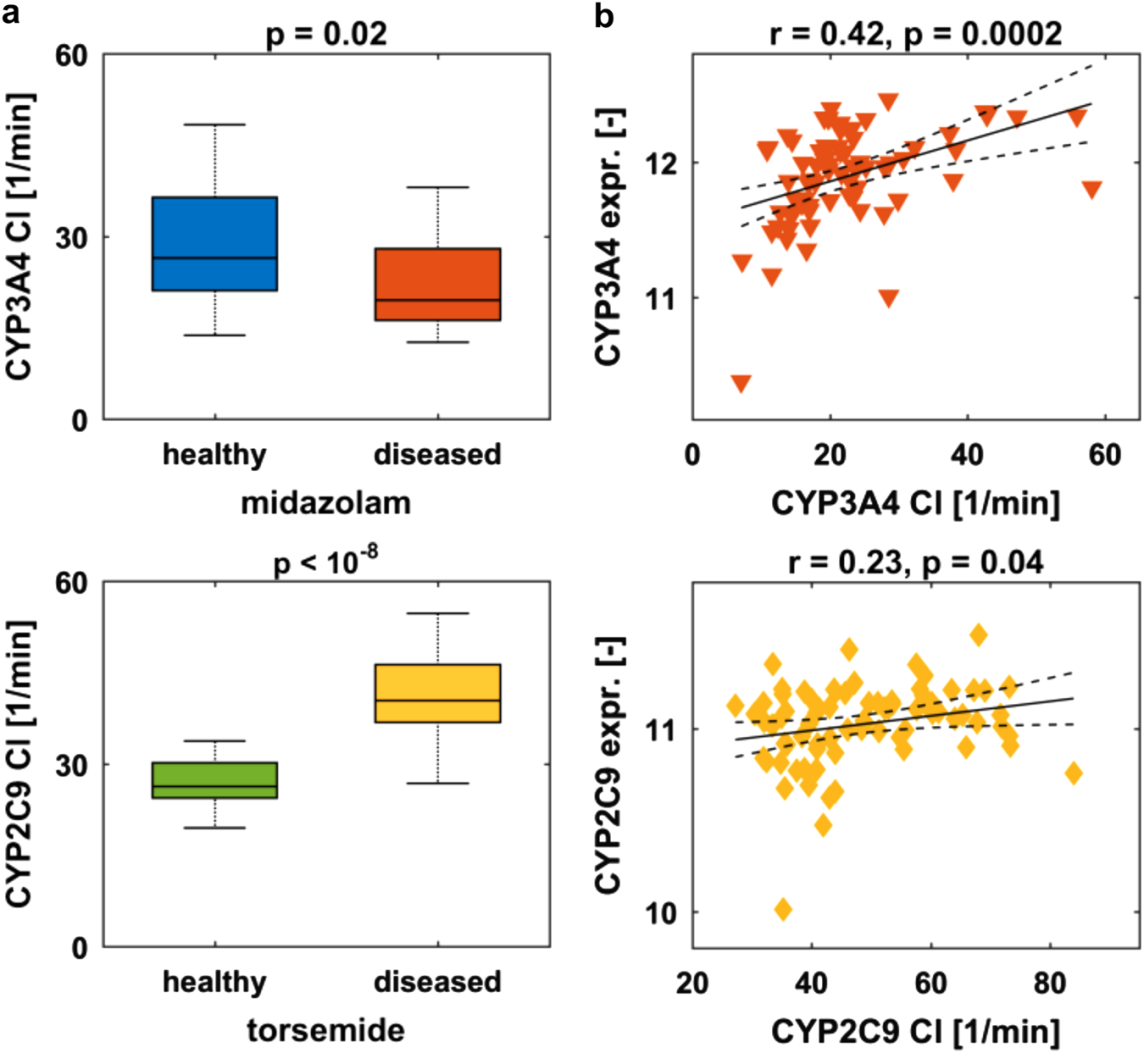
Relationships between levels of measured enzyme expression and model-assessed enzyme-mediated clearance. **a** Comparison of the specific CYP3A4-mediated clearance (CYP3A4 Cl) for midazolam, and that of CYP2C9 (CYP2C9 Cl) for torsemide, respectively, in the cohorts of 20 healthy individuals and 20 obese patients. Boxplots are defined corresponding to Fig. 2. **b** Correlation of specific hepatic clearance with expression levels of the indicated enzyme in all 79 diseased patients. Data are shown together with regression line and confidence interval for regression line (*dashed line*)

The mechanistic evaluation of PBPK models in the Bayesian-PBPK analyses allows the characterization of specific biological processes relevant for drug PK in detail. In particular, we specifically assessed the acquired knowledge in enzyme-mediated hepatic clearance, since changes in enzymatic activity in obese patients are still a matter of debate.^32^ Our analyses revealed that the hepatic metabolic clearance differs statistically significantly between the subjects of the healthy and obese cohorts. In particular, CYP3A4-mediated clearance decreased in the obese cohort (*p* = 0.02), while CYP2C9-mediated clearance increased (*p* < 10^−8^) (Fig. 6a). One of the unique features of our clinical investigation was the availability of liver biopsies from each patient. This provided the opportunity to compare assessed information from the PBPK model with underlying molecular alterations measured in corresponding patient material, i.e., expression data of the metabolizing enzymes. Interestingly, a significant correlation could be observed between CYP3A4-mediated clearance in the PBPK model and corresponding expression data of CYP3A4 (*r* = 0.51, *p* = 0.02). For torsemide, however, a direct correlation of specific hepatic clearance to the expression of CYP2C9 was not found in the cohort of 20 obese individuals. Since the small sample size might hamper the identification of statistically significant features, further Bayesian-PBPK analyses were additionally performed for midazolam and torsemide, taking into account all 79 diseased patients (59 obese patients together with the 20 individuals with body mass index (BMI) < 30, where also liver biopsies were available). The subsequent correlation analyses of hepatic clearance with corresponding expression data of CYP3A4 and CYP2C9, respectively, revealed indeed significant correlations (*r* = 0.42, *p* = 0.0002 and *r* = 0.23, *p* = 0.04, respectively) for both drugs (Fig. 6b).

Based on these relationships in the cohort of diseased patients, we next correlated hepatic clearance and expression data, respectively, with body weight and disease progression markers such as steatosis and NAS. Statistically significant correlations of hepatic CYP3A4-mediated clearance with body weight, steatosis, and NAS were observed for midazolam (Fig. S5A). For torsemide, only a correlation of hepatic CYP2C9-mediated clearance to body weight was statistically significant. Finally, the same analyses were performed with expression data of the CYP3A4 and CYP2C9 genes that have been measured in liver biopsies. The same trend was observed for all correlations, but only the relationship between CYP3A4 expression and steatosis showed a statistically significant correlation (Fig. S5B).

## Discussion

Drug development has been efficiently supported by the development of systems approaches that focus on the integration of experimental data, available knowledge and mathematical models and that translate gained knowledge from preclinical stages to the clinics.^33, 34^ However, a previous shortcoming has been the lack of sufficient techniques to not only collect but also to connect the data which is obtained along and across species, scales, and study designs.^35^ A further limitation is a missing portability of existing models and approaches.^36^ To overcome this issue, e.g., mechanistic and systems models have been identified as a possible means to generate a better understanding of biology and pathophysiology.^3^

In the present work we introduce a translational systems pharmacology workflow that addresses above challenges by combining drug cocktail probing with mechanistic PBPK modeling and Bayesian statistics. In three learning steps, initial information about drug physicochemistry and individual physiology information was used together with experimental PK data to acquire knowledge about a reference probe drug (midazolam) and a candidate probe drug (torsemide), respectively, both in a healthy population and an obese patient cohort.

Notably, the information acquired in the Bayesian-PBPK analyses allows to consider both individualized PK profiles as well as population simulations which were both used for qualification of each step of the translational workflow (Figs. 3, 4). Each learning step demonstrated significant improvement in the agreement between simulations and observed data, compared with the initial mean value model for an average patient. Ultimately, this allowed the consideration of both individualized PBPK models as well as a the quantification of the interindividual variability and its specific physiological sources, which are both highly valuable information for decision-making in pharmaceutical research programs.^3, 33^

By our iterative learning process we were able to show continuous acquisition of knowledge, especially a large gain of information was achieved in the characterization of the pathophysiology of the obese patient cohort (Fig. 5). The acquired knowledge about the candidate probe drug torsemide and the pathophysiological changes of the reference probe drug midazolam was then used for successful prediction of the population PK of torsemide in the obese population. Note that accuracy of the prediction could have been increased by including more than the 20 chosen individuals into each learning step. However, as we aimed for a translation from clinical phase I to phase II, we used a number of individuals typical for these phases of clinical development.

The retrospective analysis of torsemide PK in the obese population showed that the prediction could have been improved by integration of further enzyme-specific information for CYP2C9 in the diseased population (Table 2). Hence, the incorporation of several learning steps including studies with other reference compounds that are metabolized via CYP2C9 in the population of interest could improve the prediction of the candidate compound. In general, the ability to make predictions of future experiments and results by the integrative concept of our translational approach is of importance in drug development, as drug failure that emerges in the late phases of clinical development is often due to insufficient efficacy.^37^ The predicted population simulations together with the identified sources of PK variability could support among others evaluation steps in clinical development, where the challenge is to make decisions about project termination as early as possible.^33, 37^

A further advantage of the PBPK models integrated in our translational approach is the detailed mechanistic structure, which allowed to derive information from PK data on a functional level. Interestingly, we discovered that metabolic clearance of midazolam decreased significantly for obese patients compared with healthy volunteers, while the CYP2C9-mediated clearance of torsemide increased significantly. Thus, our results support clinical findings,^38, 39^ as hepatic clearance of midazolam showed significant inverse relationships to body weight, steatosis, and NAS in the overall diseased population (Fig. S5A).

Interestingly, the same correlation reported for hepatic clearance could be shown for expression data of CYP3A4 and CYP2C9, which confirms that the above-mentioned model-based pathophysiological characterization is feasible for specific parameters by our translational approach. These findings could pave the way for the application of comprehensive PBPK/PD systems pharmacology approaches that describe on-target drug exposure and resulting PD effects. In particular, the extension of PBPK models with PD models that mechanistically describe drug effects or biomarker relationships could lead to an improved predictability of PD effects. These models could then be used for target identification and a better understanding of the mode of action of a new drug candidate. However, challenges are, e.g., the lack of sufficient experimental data, incomplete mechanistic knowledge of the underlying processes, or the different time scales of such multi-scale modeling systems.^35, 36, 40, 41^

The presented case study is a proof-of-concept that bridging of phase I and II studies in the clinical development process is feasible. A continuous use of such translational modeling approaches can therefore support the extraction and transfer of knowledge throughout the different phases of drug development (Fig. S6). This is expected to facilitate the streamlining of future clinical studies, since extensive information is already available from prior analyses, either through the literature or through in-house databases of compounds from inventor companies.

## Materials and methods

### Study design

Two open label single-dose PK studies, conducted in healthy volunteers patients (EudraCT 2011-002291-16, ClinicalTrials.gov NCT01788254) and diseased patients (EudraCT 2012-000447-27), received regulatory approval by the German Federal Drug Administration (BfArM) and have been given favorable opinion by the local ethics committees of the University of Tuebingen and Kiel, respectively. The studies were operated in accordance with the principles of the Declaration of Helsinki and the German Drug Law (AMG). All study participants gave written informed consent before inclusion.

Female and male healthy volunteers aged 18–56 years (mean BMI: 23.5 kg/m^2^) were eligible for inclusion. Absence of diseases relevant to safety and PK was established based on a detailed medical history, thorough physical examination, electrocardiogram, and routine chemistry and hematological parameters within the institutional normal reference range. Female and male patients aged 20–77 years (mean BMI: 47.3 kg/m^2^) undergoing visceral surgery who were also scheduled for liver biopsy and otherwise in good clinical condition at the discretion of the responsible physician and investigator were included in the patient study (Table S1). Patients with signs of decompensated disease were not eligible for inclusion.

The study medication was applied after an overnight fast. The patients were studied on the day before visceral surgery. All probe drugs were administered orally as a pharmacologic cocktail containing 1 mg midazolam, 5 mg codeine, 0.25 mg torsemide, 5 mg pravastatin, 2.5 mg talinolol, and 50 mg [13C3]caffeine with 200 ml of tap water. Blood samples were drawn in EDTA-containing tubes before drug intake and after 0.5, 1, 1.5, 2, 3, 4, 6, and 8 h. Samples were centrifuged (10 min, 4 °C, 4000 rpm) and plasma was stored at −20 °C until analysis. Urine was collected for 8 h in two 4-h intervals. After recording the volume, samples were withdrawn and stored at −20 °C until analysis.

The liver biopsies were taken surgically the following day in the course of intervention and preserved in liquid nitrogen within 60 s after resection (see Supplementary Material for more information about specific methods).

### Quantification of midazolam, 1′-hydroxymidazolam, torsemide, and hydroxytorsemide in plasma

Plasma samples (250 µl) were mixed with 500 µl of acetate buffer (0.1 M, pH 5) and 10 µl of internal standard mixture. Samples were extracted with diethyl ether, and the organic phase evaporated to dryness in a stream of nitrogen. The residue was dissolved in 75 µl of acetonitrile:water 1:2 (v/v), and 5 µl of the supernatant was used for LC–MS–MS analysis as described previously.^42^

### Genotyping for CYP3A4, CYP2C9, and CYP3A5 alleles

High-quality genomic DNA was isolated from whole blood using QIAamp DNA Blood Mini Kit (Qiagen, Hilden, Germany). Genotype analyses of healthy individuals (*n* = 103) and patients (*n* = 80) for CYP3A4*22, CYP3A4*1B, CYP2C9*2, CYP2C9*3, and CYP3A5*3 were performed with predeveloped TaqMan Assays (Life Technologies, Foster City, CA, USA), according to the manufacturer’s protocol on a 7900 Real-Time PCR System (Life Technologies, Foster City, CA, USA), and data were analyzed with the SDS software. Genotype frequencies of both cohorts were in Hardy–Weinberg equilibrium.

### NAFLD activity score

The proposed NAS is the unweighted sum of steatosis, lobular inflammation, and hepatocellular ballooning scores, as developed in Kleiner *et al*.^30^ NAS of ≥5 is assigned with a diagnosis of NASH, 3–4 is assigned as “mild steatosis”, and biopsies with scores <3 are assigned as “not NASH”.

### Expression analysis

For homogenization of 5–10 mg frozen tissue and subsequent nucleic acid isolation, tubes with 1.4 mm ceramic beads (Precellys, Villeurbanne, France) and the AllPrep DNA/RNA Mini Kit (QIAGEN, Hilden, Germany) were used. Gene expression analysis using the HuGene 1.1 ST gene (Affymetrix, Santa Clara, CA, USA) was performed, according to the manufacturer’s protocols.

### PBPK models

Both the organs and physiological processes are explicitly represented in PBPK models and parametrized by physiological information provided in the PBPK software tools. So-called distribution models, which quantify passive permeation of tissue and cell membranes by a drug, are parametrized by drug physicochemistry involving lipophilicity or molecular weight.^43–46^ Finally, information on the relative tissue-specific expression of metabolizing enzymes or transport proteins is used to model active transport and metabolization of the drug.^47^ The systematic integration of patient physiology and drug physicochemistry into a PBPK model allows for a comprehensive description of the physiological processes underlying the concentration of the drug in the plasma and various tissues and organs over time.

### Midazolam PBPK model

A combined PBPK model for midazolam and OH-midazolam was created incorporating available literature information, which is described in the Supplementary Material. Three active processes described via Michaelis–Menten kinetics were integrated into the model: the metabolization of midazolam to OH-midazolam via CYP3A4; the glucuronidation of OH-midazolam via UGT1A4; and the active transport of midazolam via P-glycoprotein (PGP). The relative expression of the active form of each enzyme in each organ was obtained from the expression database integrated into the software. CYP3A4 is expressed mainly in the liver and the intestinal compartments; UGT1A4 is present in the kidneys, liver, and small intestine; PGP is expressed at high levels in all three organs. The glucuronidation of midazolam was neglected, as was its metabolization into minor metabolites via CYP3A4 and CYP3A5. The parameters provided in Table 3 are sufficient to parameterize the PBPK models for midazolam and OH-midazolam as such representing a mean individual (Model file S1).^19^

**Table 3:** Parameterization of the midazolam mean value model

Molecule	Parameter	Value	Unit
Midazolam	Fraction unbound	3	%
Midazolam	Lipophilicity	3.6	[–]
Midazolam	Molecular weight	325.77	g/mol
Midazolam	Intestinal permeability	5.55E-04	cm/min
Midazolam	Solubility at reference pH	0.03	mg/ml
Midazolam	CYP3A4 kcat	0.1	1/min
Midazolam	CYP3A4 Km	2.1	µmol/l
Midazolam	ABCB1 kcat	177.64	1/min
Midazolam	ABCB1 Km	40.45	µmol/l
OH-midazolam	Fraction unbound	10	%
OH-midazolam	Lipophilicity	3.13	[–]
OH-midazolam	Molecular weight	341.8	g/mol
OH-midazolam	UGT1A4 kcat	10	1/min
OH-midazolam	UGT1A4 Km	1.41	µmol/l

### Torsemide PBPK model

A combined PBPK model for torsemide and the metabolite M1 (OH-torsemide) was created using literature information, which is described in the Supplementary Material. Since torsemide and OH-torsemide are highly bound to plasma proteins, the exchange between plasma and interstitial space is assumed to be not instantaneous, i.e., the capillary endothelium constitutes a true barrier for this drug. This was taken into account by introducing an endothelial barrier. Values for endothelial permeability (default value for small molecules: 100 cm/min) in all organs were scaled with a factor of 0.001, except for liver. Liver permeability was scaled with a factor of 0.1 to account for endothelial fenestration in this organ. Metabolism via CYP2C9 was integrated as an active second-order Michaelis–Menten process for the production of M1 from torsemide, and of M5 from M1; the expression database integrated into PK-Sim was used to obtain the relative expression profile for the enzyme in each organ. Two independent kinetics equations were used since the dissociation constants Km and vmax would not necessarily be the same for both metabolization steps. For the sake of simplicity, and due to a lack of experimental data, the production of M3 was neglected. Transport via OATP1B1 was also integrated into the model. It was assumed that both torsemide and OH-torsemide are transported via OATP1B1, but with different kinetics, similar to the assumption for CYP2C9. Two renal clearance processes were also integrated into the model, one for each of the considered drugs. Michaelis–Menten kinetics were used so that potential nonlinearities could be described. The parameters provided in Table 4 are sufficient to parameterize the PBPK models for torsemide and OH-torsemide as such representing a mean individual (Model file S2).^19^

**Table 4:** Parameterization of the torsemide mean value model

Molecule	Parameter	Value	Unit
Torsemide	Fraction unbound	1.25E-01	%
Torsemide	Lipophilicity	2.023	[–]
Torsemide	Molecular weight	348.8	g/mol
Torsemide	Intestinal permeability	1.66E-05	cm/min
Torsemide	CYP2C9 kcat	20	1/min
Torsemide	CYP2C9 Km	1	µmol/l
Torsemide	OATP1B1 kcat	30	1/min
Torsemide	OATP1B1 Km	1	µmol/l
Torsemide	Renal clearance	0.0013	l/min/kg
OH-torsemide	Fraction unbound	1.91E-01	%
OH-torsemide	Lipophilicity	2.139	[–]
OH-torsemide	Molecular weight	364.42	g/mol
OH-torsemide	CYP2C9 kcat	70	1/min
OH-torsemide	CYP2C9 Km	1	µmol/l
OH-torsemide	OATP1B1 kcat	50	1/min
OH-torsemide	OATP1B Km	1	µmol/l
OH-torsemide	Renal clearance	0.007	l/min/kg

### Statistical analysis

A previously established Bayesian-PBPK analysis was used for individual steps of the presented translational approach.^24^ The objective of the Bayesian-PBPK analysis is the identification of a large number of physiological and substance-specific parameters. In Bayesian statistics, parameter uncertainty can directly be derived in form of probability distributions. Furthermore, the Bayesian formulation integrates prior information (i.e., initial knowledge) about unknown parameters into the estimation process. The prior information is then combined with the information extracted from the experimental data in the so-called posterior distribution (i.e., acquired knowledge). For most parameters, informative prior distributions such as normal and lognormal distributions can be defined. However, when the available information is vague, so-called uninformative (e.g., uniform) distributions are defined. Nevertheless, for each parameter, the lower and upper boundaries that are specified correspond to biological information.

Distributions of these physiological parameters were refined by using a Bayesian framework in combination with mixed-effects modeling.^24, 48, 49^ The hierarchical structure of the mixed-effects model separates the individual level from the population level. At the individual level, the experimental data were provided for each individual and the PBPK model describing the experimental data is specifically parameterized for the individual. At the population level, all individual parameters are assigned a population distribution and the population distribution itself was identified in the Bayesian-PBPK analysis. The resulting complex distribution is identified via a Markov chain Monte Carlo (MCMC) approach (see also Supplementary Methods).^50, 51^

For each application of the Bayesian-PBPK analysis, one long MCMC sample was generated by drawing 150,000 samples from of the posterior distribution. The Gelman and Rubin convergence measure ($\hat{R}$) was calculated to determine the point of convergence of the chain of samples, since only the converged portion of the chain represents the posterior distribution.^52, 53^ Convergence was determined after 50,000 iterations. From the remaining 100,000 iterations, a random and independent subsample of 500 was identified for the analyses and simulations that are presented (see also Supplementary Methods).

The Bayesian-PBPK analyses were qualified by performing population simulations that compare the assessed interindividual variability with experimental data from individuals who were not integrated into the analyses. Therefore, a virtual population was created based on the subsample from the posterior distribution. Each virtual individual assessed in this analysis was created randomly, using age, body height, body weight, and BMI within the overall range of the study population (Table 1). To create a new virtual individual, a random parameter vector was drawn out of the updated population distributions using the posterior subsample. The population distributions themselves were created by taking the maximum posterior estimates of the population parameters (mean value and standard deviation). For a detailed description of how a new individual is sampled,^24^ correlation analyses were performed by calculating Pearson’s linear correlation coefficient. Significance of the difference between mean values was tested using a two-sample *t*-test. Significance level was 0.05.

### Implementation

The translational approach including the Bayesian-PBPK analyses was implemented in Matlab® (version R2013b; MathWorks, Natick, MA). The PBPK models were created using the software tools PK-Sim® (version 5.3.2) and MoBi® (version 3.3.2). PK-Sim and MoBi are both part of the Computational Systems Biology Software Suite, which is a commercial software package from Bayer AG, Leverkusen, Germany, and for which academic licenses are available free of charge. It further consists of R and Matlab Toolboxes, which represent interfaces to MoBi, such that parameterization and simulation of a PBPK model created within PK-Sim is possible also in external software. This allows to integrate the simulation into complex workflows. Computation was performed on a computer cluster running the SUSE Linux Enterprise Server 11 SP3 operation system. The cluster consists of 36 knots whereby each knot consists of two CPU containing 16 cores. Model evaluations were parallelized such that each individual was evaluated on a single core.

## Electronic supplementary material

Supplementary Information
