# Supplementary material for: Translational learning from clinical studies predicts drug pharmacokinetics across patient populations
Source: NPJ Syst Biol Appl. 2017 Mar 28;3:11. doi: 10.1038/s41540-017-0012-5 (PMC5460240; doi:10.1038/s41540-017-0012-5)
Supplement: Supplementary file 1 — Supplementary Information [file 41540_2017_12_MOESM1_ESM.docx]

# ---- Supplementary Information ---

**Translational learning from clinical studies predicts drug pharmacokinetics across patient populations**

**Authors:** Markus Krauss^1,^*, Ute Hofmann^2,^*, Clemens Schafmayer^3,^*, Svitlana Igel^2,^*,^,^ Jan Schlender^1^, Christian Mueller^4^, Mario Brosch^5,6^, Witigo von Schoenfels^3^, Wiebke Erhart^3^, Andreas Schuppert^7,8^, Michael Block^1^, Elke Schaeffeler^2^, Gabriele Boehmer^9^, Linus Goerlitz^4^, Jan Hoecker^3^, Joerg Lippert^10^, Reinhold Kerb^2,#^, Jochen Hampe^5,6,#^, Lars Kuepfer^1,#,§^, Matthias Schwab^2,9,11,#^

* contributed equally

# contributed equally

§ corresponding author

**Affiliations:**

^1^Systems Pharmacology, Bayer AG, 51368 Leverkusen, Germany.

^2^Dr. Margarete Fischer-Bosch Institute of Clinical Pharmacology and University of Tuebingen, 70376 Stuttgart, Germany.

^3^Department of General Surgery and Thoracic Surgery, University Hospital Schleswig-Holstein, 24105 Kiel, Germany.

^4^Applied Mathematics, Bayer AG, 51368 Leverkusen, Germany.

^5^Department of Medicine I, University Medical Center Dresden, Technical University Dresden, 01307 Dresden, Germany

^6^Department of Internal Medicine I, University Medical Center Schleswig-Holstein, 24105 Kiel, Germany.

^7^Technology Development, Bayer AG, 51368 Leverkusen, Germany.

^8^Joint Research Center for Computational Biomedicine, RWTH Aachen University, 52074 Aachen, Germany.

^9^Department of Clinical Pharmacology, University Hospital Tuebingen, 72076 Tuebingen, Germany.

^10^Clinical Pharmacometrics, Bayer Pharma AG, 13353 Berlin, Germany.

^11^Department of Pharmacy and Biochemistry, University of Tuebingen, 72074 Tuebingen, Germany.

# Supplementary Materials

Materials and Methods

Fig. S1: Dependence on body weight

Fig. S2: Individual model simulations for all parent drugs.

Fig. S3: Individual model simulations for all metabolites.

Fig. S4: Comparison of medians of population simulations for torsemide and OH-torsemide in the obese cohort.

Fig. S5: Correlation of model output and measurements with disease progression markers for the Bayesian-PBPK analyses using all individuals of the diseased patient cohort.

Fig. S6: Iterative administration of the translational approach in the clinical drug development process.

Table S1: Co-morbidities and co-medication of the diseased population

Table S2: Summary of statistics for key PK parameters of midazolam and torsemide

Table S3: Genotype frequencies in healthy individuals and patients

Table S4: Quantitative assessment of PK prediction

Table S5: Initial and acquired distributions for the Bayesian-PBPK analysis with midazolam in healthy individuals.

Table S6: Initial and acquired distributions for the Bayesian-PBPK analysis with torsemide in healthy individuals.

Table S7: Initial and acquired distributions for the Bayesian-PBPK analysis with midazolam in obese patients.

Table S8: Initial and acquired distributions for the Bayesian-PBPK analysis with torsemide in obese patients.

Model files S1 and S2: Mean model of midazolam (“MidazolamMeanValueModel_forPaper.mbp3”) and Mean model of torsemide (“TorsemideMeanValueModel_forPaper.pksim5”) can be provided upon reader’s request.

Materials and Methods

## Additional information to study design

Healthy volunteers were not allowed to take any drugs other than the test drugs (and oral contraceptives) from 7 days prior to the study day through 24 hours after application of the probe drugs. Patients were excluded if they used any medication known to interact with the probe drugs. All study participants were required to abstain from alcohol or caffeine (coffee, tea, chocolate) from 48 hours before until 12 hours after dosing. Starting one week before and throughout the study, the following foods were not allowed: grapefruit, chinine-containing beverages, star fruit (carambola), broccoli, any cabbage, watercress, char-grilled food, mustard.

Both clinical studies applied identical study medication (same batches) and study procedures. Packaging and labeling of torsemide, talinolol, pravastatin, and [13C3]caffeine was performed by the pharmacy of the University Hospital Ulm (Ulm, Germany). Capsules containing the required amount of the respective drug were manufactured from commercially available tablets (torsemide: Torem RR 2.5 mg tablets, Berlin-Chemie (Berlin, Germany), pravastatin: Pravastatin Hexal 10 mg tablets, Hexal (Holzkirchen, Germany), talinolol: Cordanum 50 mg film-coated tablets, AWD.pharma (Radebeul, Germany). For the 13C-caffeine test, [trimethyl-13C3] caffeine (99% 13C), obtained as powder from Cambridge Isotope Laboratories (Cambridge, MA), was packaged in 50 mg capsules. Codeine (Codeintropfen CT, 1 mg/drop, CT Arzneimittel, Berlin, Germany) and midazolam (Midazolam ratiopharm, 2 mg/ml, Ratiopharm, Ulm, Germany) were used as commercially available packages of liquid formulations.

## Midazolam

Midazolam is a benzodiazepine derivative that is used mainly as sedative. It is metabolized almost exclusively via cytochrome P450 3A4 (CYP3A4). Metabolization takes place in the liver as well as in the intestine. The main metabolite is 1’-hydroxymidazolam (OH-midazolam), which is also pharmacologically active. Two other metabolites are formed via CYP3A4 and CYP3A5, but only in small proportions (3% and 1%, respectively). The bioavailability of orally administered midazolam is extremely variable and, due to first-pass metabolism in the intestinal tract, the rate of formation of OH-midazolam is higher after oral vs. intravenous administration. The parent drug and metabolite are both glucuronidated via Uridine 5'-diphospho-glucuronosyltransferase 1A4 (UGT1A4), with about 75% of the administered dose being renally excreted as glucuronidated OH-midazolam (the fraction of unchanged midazolam is negligible) {Greenblatt, 1984 #211;Heizmann, 1983 #237;Mandrioli, 2008 #238}. According to DrugBank {Knox, 2011 #239}, the unbound fraction in the blood plasma is about 3%, and the lipophilicity was calculated as 3.89, although other sources provide values of between 2.9 {Vossen, 2007 #258} and 3.93 {Chauve, 2010 #240}. For OH-midazolam, lipophilicity has been determined as 3.13 {Chauve, 2010 #240}.

## Torsemide

Torsemide is a loop diuretic used to treat chronic heart failure, chronic renal failure and hypertension {Miners, 1995 #241}. It leads to an increase in urinary excretion, thereby reducing blood volume and pressure. Thus, it affects neither renal plasma flow nor the glomerular filtration rate {Knox, 2011 #239}. Compared to other loop diuretics like furosemide, torsemide has a high bioavailability (about 80 to 90%) {Neugebauer, 1988 #242}, which indicates that first-pass metabolism is low {Knauf, 1998 #243}. Torsemide is metabolized in the liver via cytochrome P450 2C9 (CYP2C9), forming three major metabolites, M1, M3 and M5. M5 is formed from M1 by oxidation {Miners, 1995 #241;Neugebauer, 1988 #242;Spahn, 1990 #244}. In addition, torsemide is metabolized by CYP2C8, although the intrinsic clearance ($v_{\max}$/$K_{m}$) by this enzyme is about an order of magnitude lower than that by CYP2C9 {Kerdpin, 2004 #245;Miners, 2000 #246}. Torsemide is actively transported via the organic anion-transporting polypeptide 1B1 (OATP1B1) {Vormfelde, 2008 #259}. The unbound fraction of torsemide in the blood plasma is less than 1%; thus glomerular filtration can be neglected as a route for renal excretion. Active secretion is therefore the main route of renal elimination. The liver metabolizes about 75 to 80% of torsemide, with only 20 to 25% remaining unchanged in the urine. In addition, 11%, 3% and 44% of the administered dose is present in the urine as M1, M3 and M5, respectively {Neugebauer, 1988 #242}.

## MCMC algorithm

We used both a gradient-based sampler, the manifold Metropolis-adjusted Langevin algorithm (mMALA) {Girolami, 2011 #260}, and an adaptive Metropolis-Hastings (MH) algorithm {Haario, 2005 #261}. Population parameters were sampled using mMALA, whereas individual physiological parameters and drug-specific parameters were sampled using adaptive MH. mMALA was not used in the latter case because it requires first and second derivatives of the posterior probabilities, and thus two additional PBPK model evaluations per parameter and iteration for calculation of the likelihood, which would not be efficient. However, the population level is influenced only by changes in prior distributions, such that no additional model evaluations are required and thus the use of mMALA is highly feasible in this case.

## Gelman and Rubin convergence criterion {Gelman, 1992 #256}

To determine $\hat{R}$, the remaining 100,000 samples were split into two chains, containing (1) iterations 50,001 - 100,000, and (2) iterations 100,001 - 150,000. An independent subsample of 500 parameter vectors from of the chain was used for the analyses and population simulations presented here.


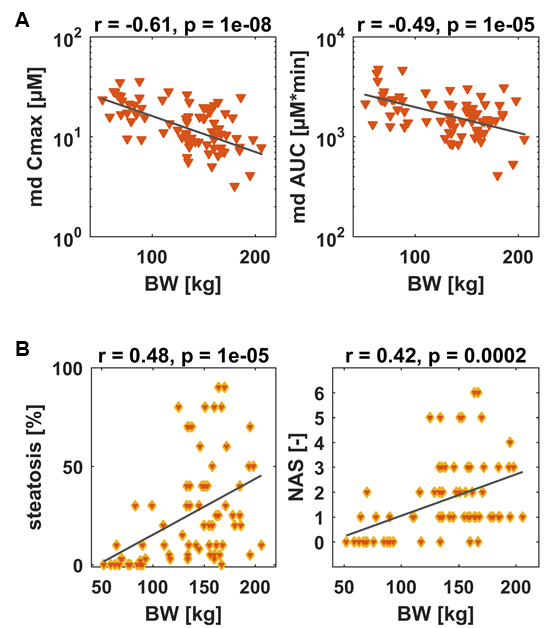


**Fig. S1: Dependence on body weight.** (A) Scatter plots and linear regression of Cmax and AUC in venous blood plasma with body weight (BW) in diseased patients. (B) Scatter plots and linear regression of steatosis of the hepatocytes and NAS from patients with body weight. Red and triangles, midazolam data from diseased population (md); yellow and diamonds, torsemide data from diseased population (td).


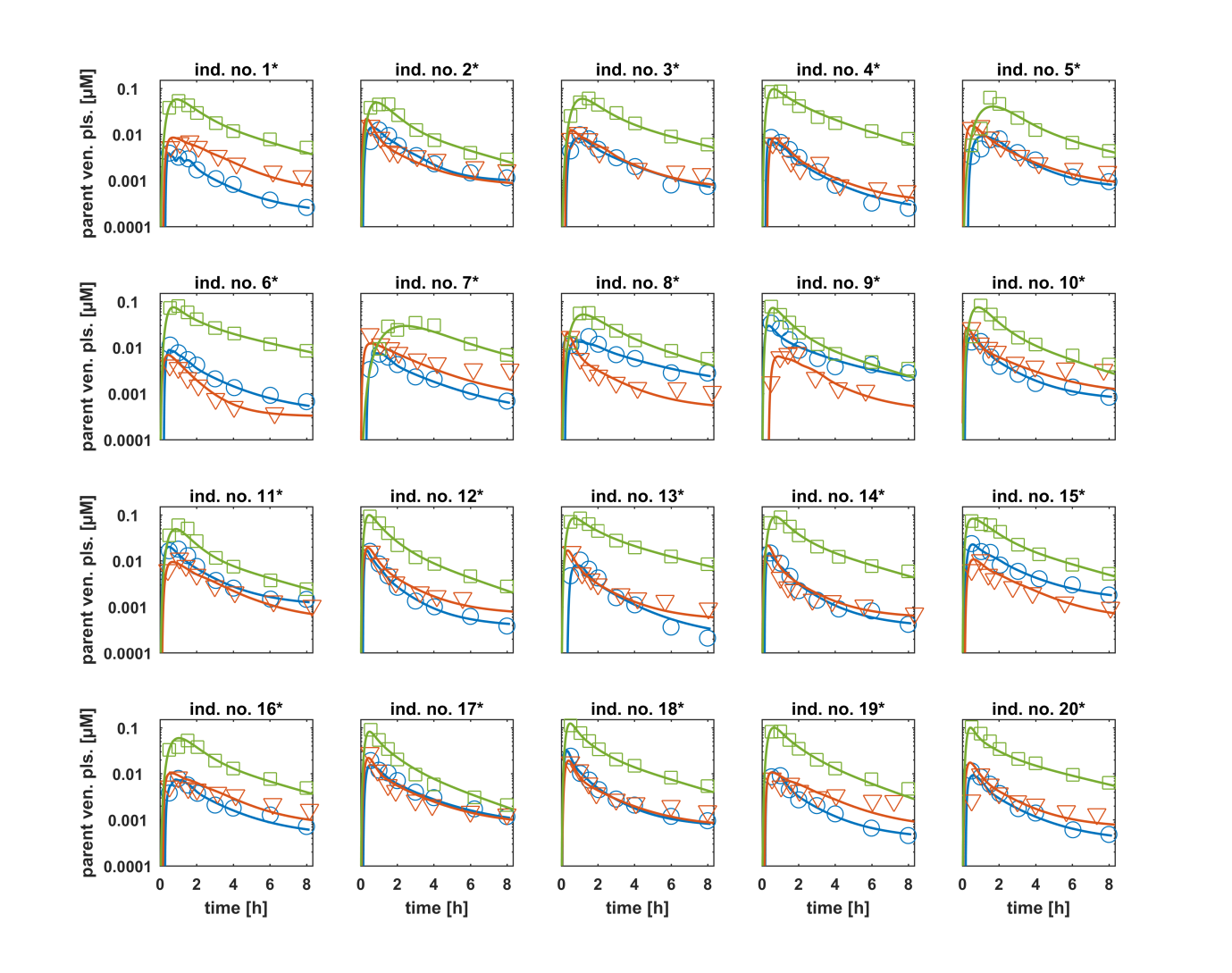


Fig. S2: Individual model simulations for all parent drugs. The simulation of the parameter vector with maximum posterior probability is shown for midazolam in healthy individuals (blue lines and circles), torsemide in healthy individuals (green lines and squares) and midazolam in obese patients (red lines and triangles). *Individuals are individuals 1-20 of the healthy and obese population, respectively.


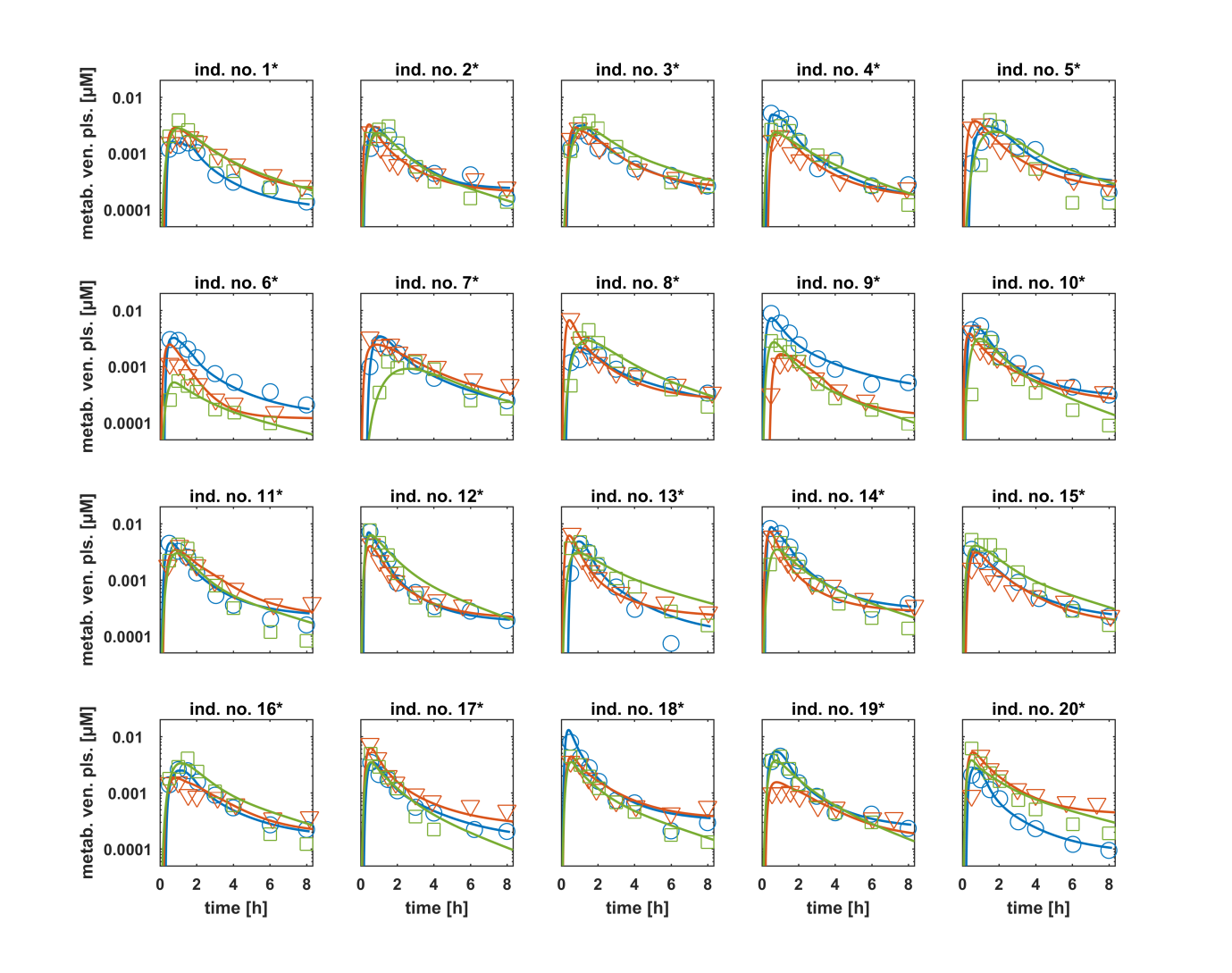


Fig. S3: Individual model simulations for all metabolites. The simulation of the parameter vector with maximum posterior probability is shown for OH-midazolam in healthy individuals (blue line and circles), OH-torsemide in healthy individuals (green line and squares) and OH-midazolam in obese patients (red line and triangles). *Individuals are individuals 1-20 of the healthy and obese population, respectively.


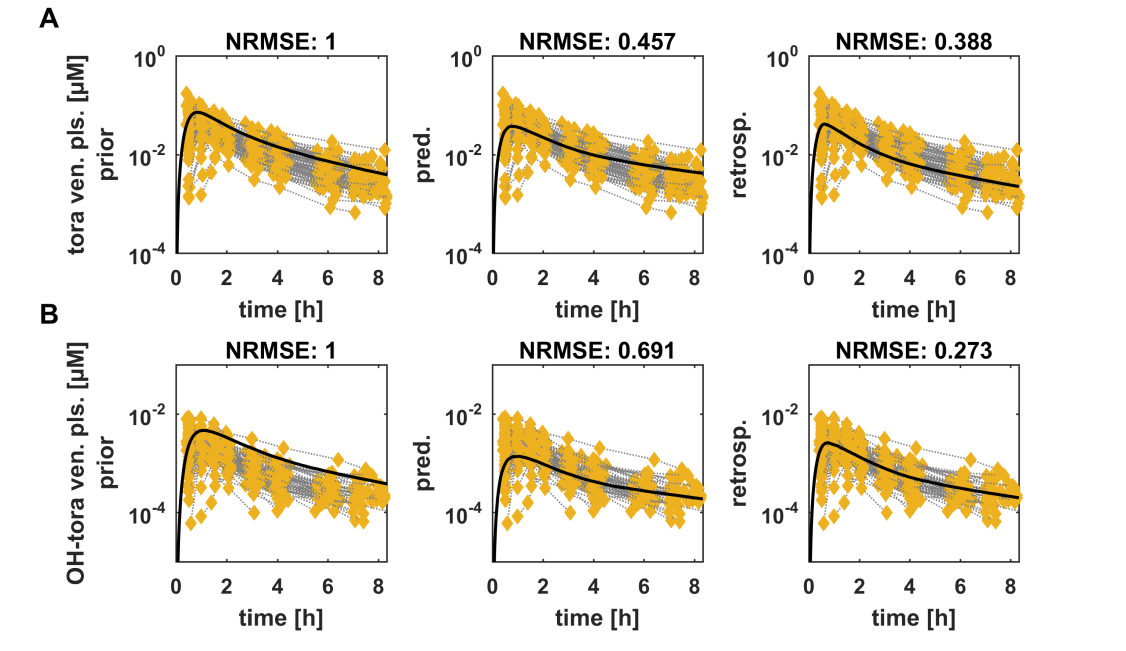


Fig. S4: Comparison of medians of population simulations for (A) torsemide and (B) OH-torsemide in the obese population. Shown are simulations (left) based only on initial knowledge, (middle) predicted by the presented translational approach, and (right) obtained after taking into account knowledge acquired from a retrospective assessment of interindividual variability for torsemide in the diseased population. Normalized root mean square errors (NRMSE) are indicated above each subfigure, normalized to largest RMSE of torsemide and OH-torsemide, respectively.


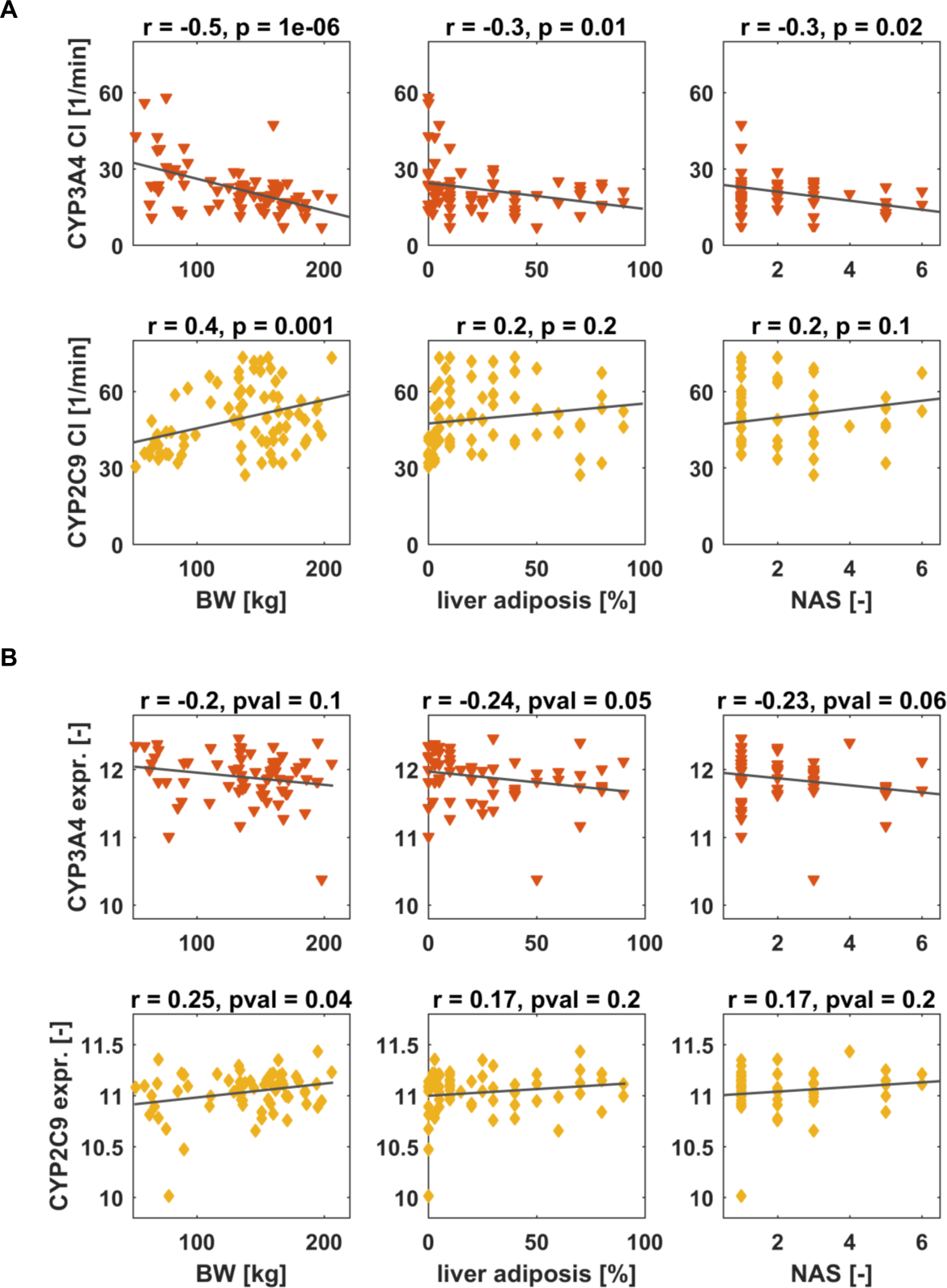


Fig. S5: Correlation of model output and measurements with disease progression markers for the Bayesian-PBPK analyses using all individuals of the diseased patient cohort. Color code: red triangles, midazolam data from diseased population; yellow diamonds, torsemide data from diseased population. (A) Correlation of the metabolic clearance by the indicated enzyme with body weight (BW), steatosis of the hepatocytes and NAS. (C) Correlation of level of expression of the indicated enzyme with body weight (BW), steatosis and NAS.


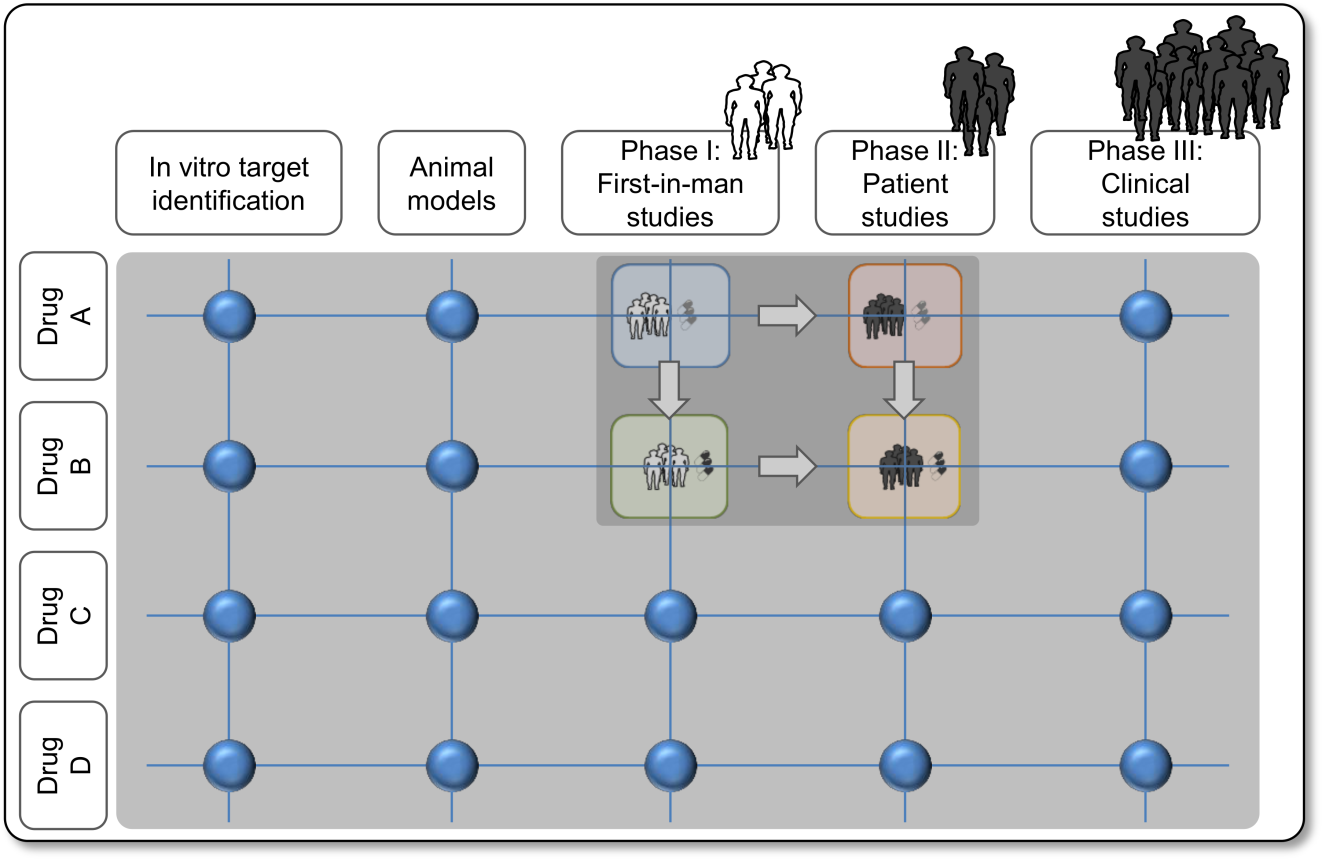


Fig. S6: Iterative administration of the translational approach in the clinical drug development process. The translational approach can be iteratively used across and within clinical development programs. As a proof-of-concept example we present translation between two drugs and between clinical phase I and II.

| Co-morbidities | proportion [%] |
| --- | --- |
| carcinoma | 21.69 |
| hypertension | 40.96 |
| adipositas | 72.29 |
| depression | 4.82 |
| diabetes | 16.87 |
|  |  |
| Co-medication | **proportion [%]** |
| hormones | 10.84 |
| Antihypertensive drugs | 20.48 |
| ACE-inhibitor | 15.66 |
| PPI | 22.89 |
| insulin | 9.64 |
| metformin etc | 8.43 |
| antidepressants | 4.82 |
| analgesics (without ASS) | 7.23 |
| chemotherapeutics | 2.41 |
| no co-medication | 43.37 |

Table S1: Co-morbidities and co-medication of the patient population

|  | Healthy | | diseased | | p-val |
| --- | --- | --- | --- | --- | --- |
|  | **GM** | **CV [%]** | **GM** | **CV [%]** |  |
| midazolam cmax [µmol/l/kg] | 0.00017 | 50 | 0.00011 | 76 | 5.43E-06 |
| torsemide cmax [µmol/l/kg] | 0.00110 | 33 | 0.00045 | 62 | 7.65E-22 |
| midazolam AUC [µmol*min/l/kg] | 0.01800 | 77 | 0.01200 | 93 | 3.03E-06 |
| torsemide AUC [µmol*min/l/kg] | 0.13000 | 62 | 0.04900 | 78 | 4.23E-27 |

Table S2: Summary of statistics for key PK parameters of midazolam and torsemide

|  |  | Healthy volunteers (n=103) | Patient cohort (n=80) |
| --- | --- | --- | --- |
| Gene | **Genotype** | **number (frequency%)** | **number (frequency%)** |
| CYP3A4 | *1/*1 | 93 (90.29%) | 65 (81.25%) |
|  | *1/*22 | 6 (5.83%) | 9 (11.25%) |
|  | *1/*1B | 3 (2.91%) | 6 (7.50%) |
|  | *1B/*22 | 1 (0.97%) | 0 (0%) |
|  |  |  |  |
| CYP2C9 | *1/*1 | 64 (62.14%) | 54 (67.50%) |
|  | *1/*2 | 21 (20.39%) | 15 (18.75%) |
|  | *2/*2 | 3 (2.91%) | 1 (1.25%) |
|  | *1/*3 | 12 (11.65%) | 9 (11.25%) |
|  | *3/*3 | 1 (0.97%) | 0 (0%) |
|  | *2/*3 | 2 (1.94%) | 1 (1.25%) |
|  |  |  |  |
| CYP3A5 | *3/*3 | 92 (89.32%) | 72 (90.00%) |
|  | *3/*1 | 11 (10.68%) | 7 (8.75%) |
|  | *1/*1 | 0 (0%) | 1 (1.25%) |

Table S3: Genotype frequencies in healthy individuals and patients

| parameter | initial geo. mean | CV [%] | acquired geo. mean | CV [%] | difference acquired/initial geo. mean [%] |
| --- | --- | --- | --- | --- | --- |
| UGT1A4 Vmax [µmol/l/min] | 45.29 | 34.1 | 223.31 | 33 | 393.07 |
| CYP3A4 Vmax [µmol/l/min] | 13.447 | 34 | 61.567 | 40 | 357.83 |
| ABCB1 Vmax [µmol/l/min] | 177.45 | 34 | 756.71 | 11.1 | 326.44 |

Table S4: Initial and acquired distributions for the Bayesian-PBPK analysis with midazolam in healthy individuals. All parameters are shown that reveal a difference of the geometric mean (geo. mean) between acquired and initial distribution being larger than 10%.

| parameter | initial geo. mean | CV [%] | acquired geo. mean | CV [%] | difference acquired/initial geo. mean [%] |
| --- | --- | --- | --- | --- | --- |
| CL renal metabolite [L/min/kg] | 0.007 | 34 | 0.011 | 35 | 57.14 |
| Fat vol. [L] | 14.88 | 42 | 20.74 | 41 | 39.38 |
| Intestinal permeability [cm/min] | 1.66E-05 | 34 | 2.18E-05 | 67 | 31.33 |
| CL renal [L/min/kg] | 0.0013 | 34 | 0.0017 | 27 | 30.77 |
| CYP2C9 Vmax [µmol/l/min] | 19.98 | 34 | 25.6 | 30 | 28.13 |
| Muscle vol. [L] | 32.35 | 10 | 24.77 | 10 | -23.43 |
| CYP2C9 Vmax metabolite [µmol/l/min] | 69.85 | 34 | 61.94 | 41 | -11.32 |

Table S5: Initial and acquired distributions for the Bayesian-PBPK analysis with torsemide in healthy individuals. All parameters are shown that reveal a difference of the geometric mean (geo. mean) between acquired and initial distribution larger than 10%.

| parameter | initial geo. mean | CV [%] | acquired geo. mean | CV [%] | difference acquired/initial geo. mean [%] |
| --- | --- | --- | --- | --- | --- |
| Muscle vol. [L] | 32.37 | 10.00 | 49.53 | 10.00 | 53.01 |
| UGT1A4 Vmax [µmol/L/min] | 228.82 | 35.00 | 119.91 | 60.00 | -47.60 |
| Fat vol. [L] | 34.27 | 15 | 48.16 | 37.00 | 40.53 |
| Fat Q spec. [1/min] | 0.02 | 5 | 0.03 | 4.00 | 27.27 |
| ABCB1 Vmax [µmol/L/min] | 756.71 | 11.1 | 922.81 | 6.00 | 21.21 |
| Gastric emptying time [min] | 17.43 | 35 | 20.61 | 38.00 | 18.24 |
| CYP3A4 Vmax [µmol/L/min] | 61.567 | 40 | 56.706 | 42.00 | -10.26 |

Table S6: Initial and acquired distributions for the Bayesian-PBPK analysis with midazolam in obese patients. All parameters are shown that reveal a difference of the geometric mean (geo. mean) between acquired and initial distribution larger than 10%.

| parameter | initial geo. mean | CV [%] | acquired geo. mean | CV [%] | difference acquired/initial geo. mean [%] |
| --- | --- | --- | --- | --- | --- |
| Muscle vol. [L] | 32.37 | 9 | 72.78 | 9 | 124.84 |
| CYP2C9 Vmax [µmol/L/min] | 25.59 | 31 | 36.64 | 30 | 43.18 |
| CL renal [L/min/kg] | 0.0016 | 25 | 0.0022 | 19 | 37.50 |
| Fat vol. [L] | 34.38 | 15 | 46.74 | 36 | 35.95 |
| CYP2C9 Vmax metabolite [µmol/L/min] | 59.71 | 43 | 43.64 | 24 | -26.91 |
| CL renal metabolite [L/min/kg] | 0.011 | 33 | 0.013 | 2 | 18.18 |
| Gastric emptying time [min] | 17.32 | 35 | 20.33 | 38 | 17.38 |
| Intestinal permeability [cm/min] | 2.17E-05 | 68 | 2.47E-05 | 77 | 13.82 |
| Spleen vol. [L] | 0.24 | 43 | 0.27 | 47 | 12.50 |
| Bone Q spec. [1/min] | 0.027 | 5.01 | 0.03 | 5 | 11.11 |

Table S7: Initial and acquired distributions for the Bayesian-PBPK analysis with torsemide in obese patients. All parameters are shown that reveal a difference of the geometric mean (geo. mean) between acquired and initial distribution being larger than 10%.
